# Supplementary material for: Molecular Mechanisms in Murine Syngeneic Leukemia Stem Cells
Source: Cancers (Basel). 2023 Jan 24;15(3):720. doi: 10.3390/cancers15030720 (PMC9913241; doi:10.3390/cancers15030720)
Supplement: Supplementary file 1 [file cancers-15-00720-s001.zip › Supplementary figures S1-S4 ML23 LSC 8.12.2022.pdf]

Supplementary information for Molecular mechanisms in Murine Syngeneic Leukemia Stem Cells

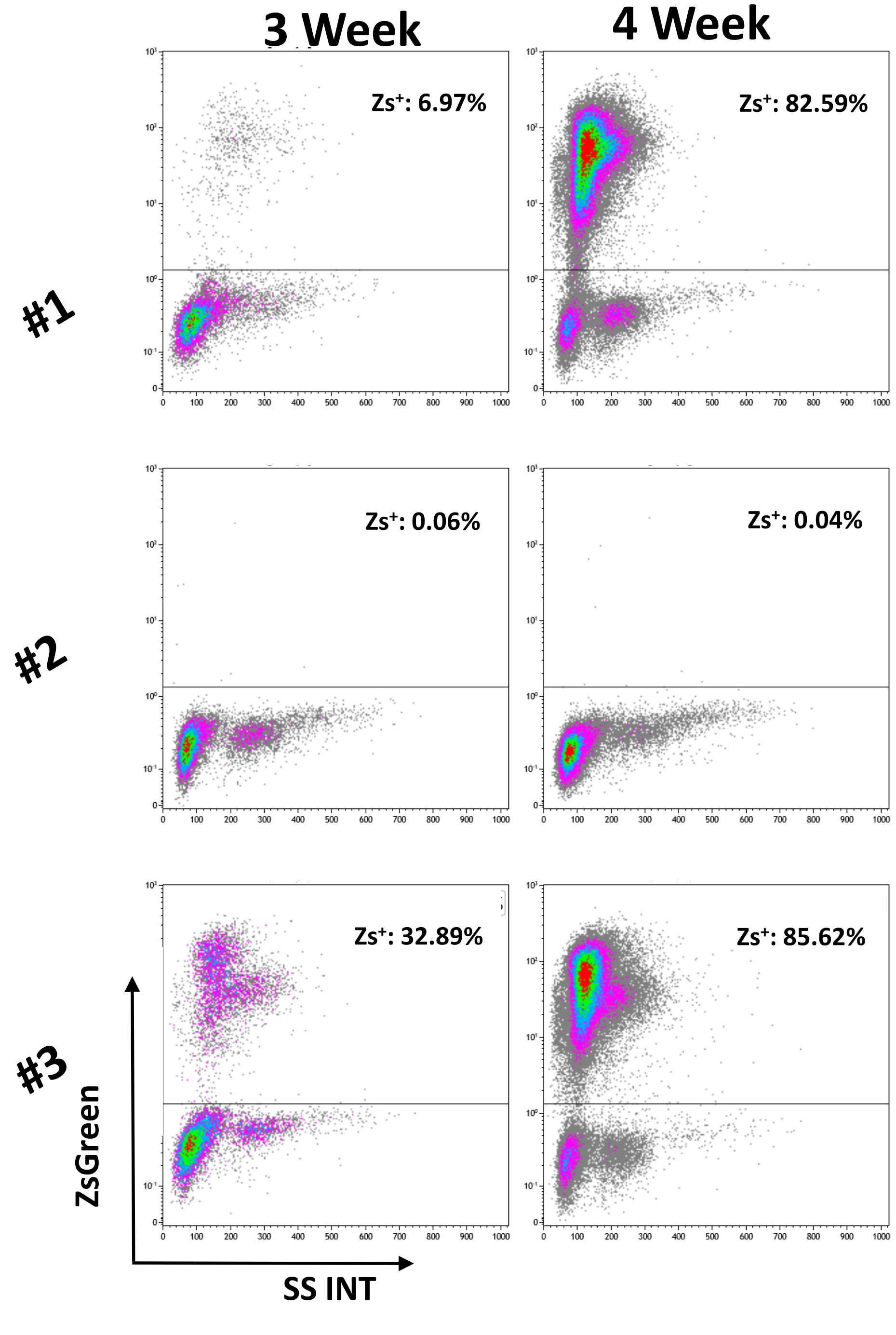

<u>Figure S1. ML23 line presents consistent and stable leukemia development:</u> Frozen BM cells from ML23 leukemic mice were defrosted and then transplanted into 3 recipients mice (5.5×10^5 cells each). The leukemic reporter ZsGreen+ (Y-axis) was initially found in the PB of 2 mice (#1 and #3) after 3 weeks. Within 4 weeks of transplantation mice #1 and #3 presented acute leukemia phenotype and high Zsgreen+ expression of PB cells.

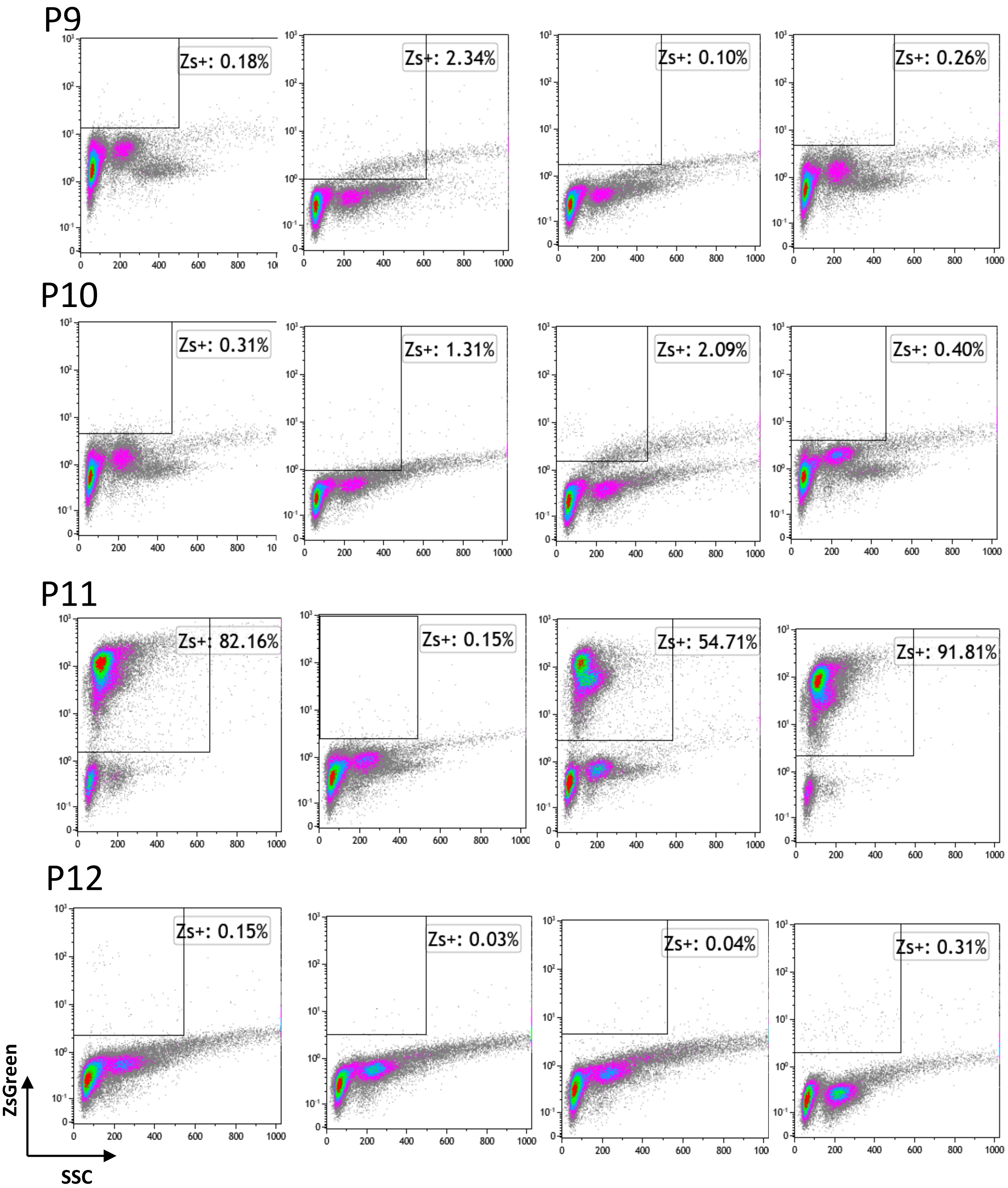

<u>Figure S2. FACS plots for ZsGreen in the different subpopulations</u> <u>of ML23:</u> FACS plots descripting the amount of ZsGreen+ expressed in each sample for each of the subpopulations. This data is a representative for 3 independent experiments.

(a)

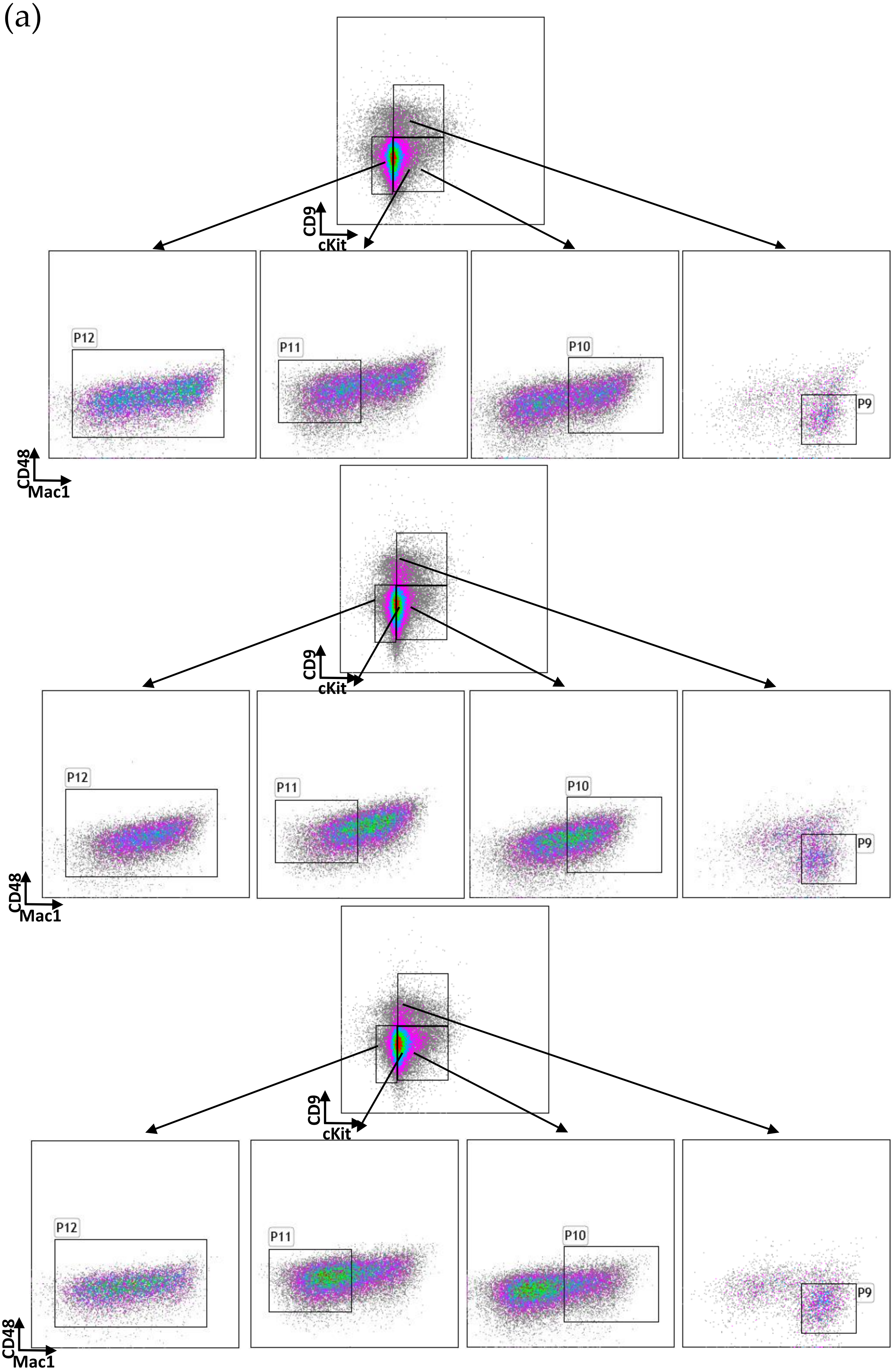

(b)

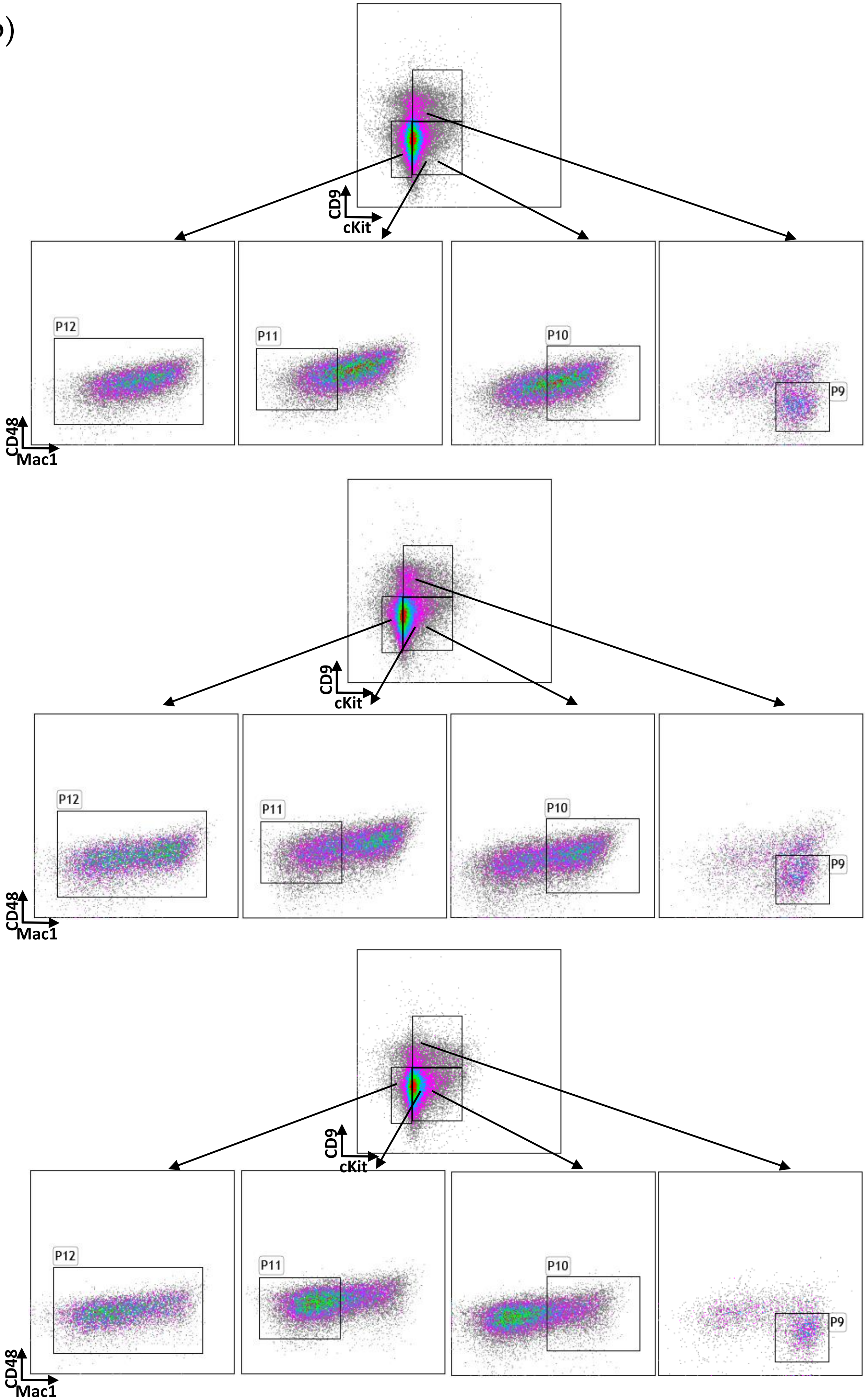
CD9
cKit
P12
P11
P10
P9
CD48
Mac1
CD9
cKit
P12
P11
P10
P9
CD48
Mac1
CD9
cKit
P12
P11
P10
P9
CD48
Mac1

(c)

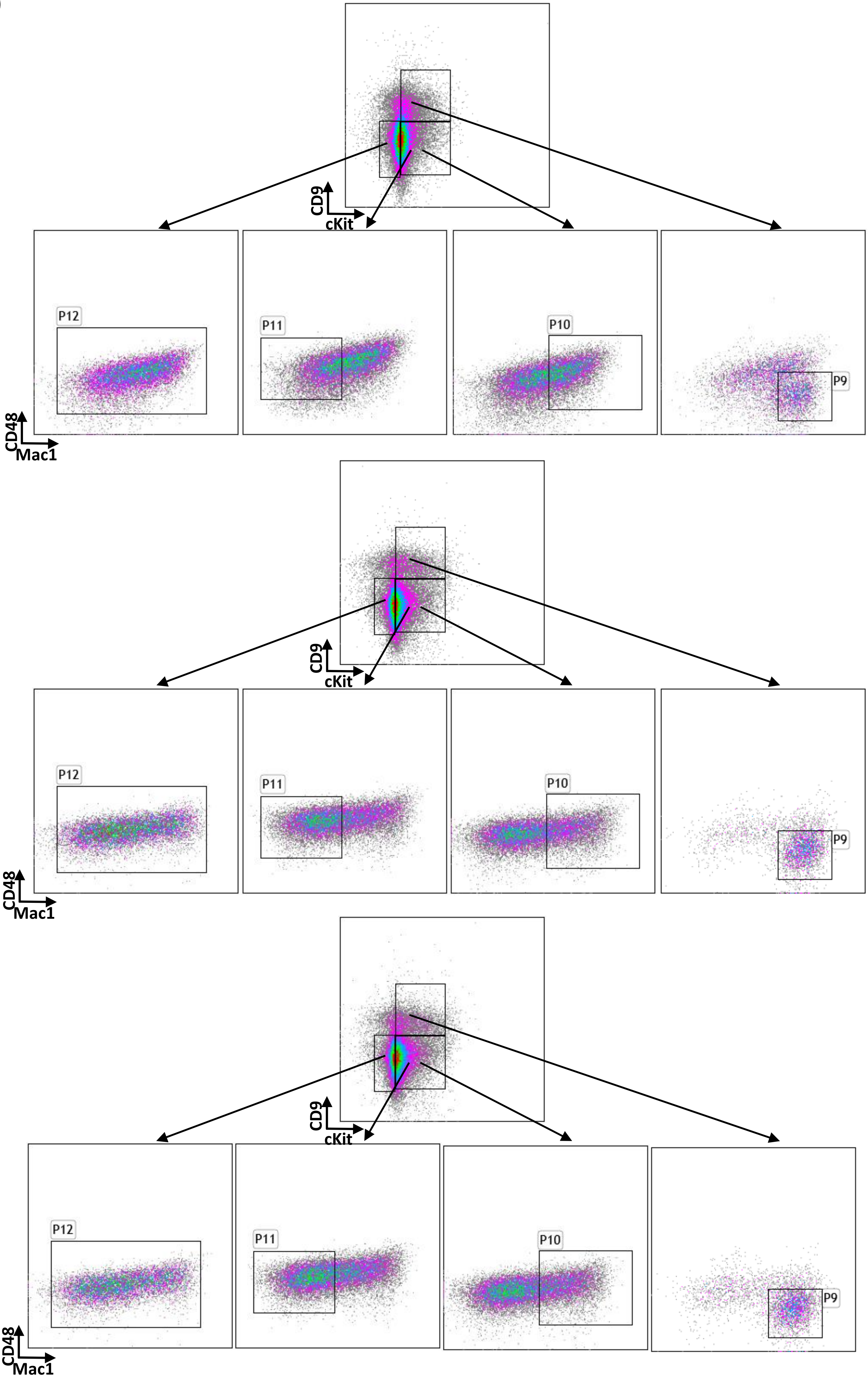
CD9
cKit
P12
P11
P10
P9
CD48
Mac1
CD9
cKit
P12
P11
P10
P9
CD48
Mac1
CD9
cKit
P12
P11
P10
P9
CD48
Mac1

(d)

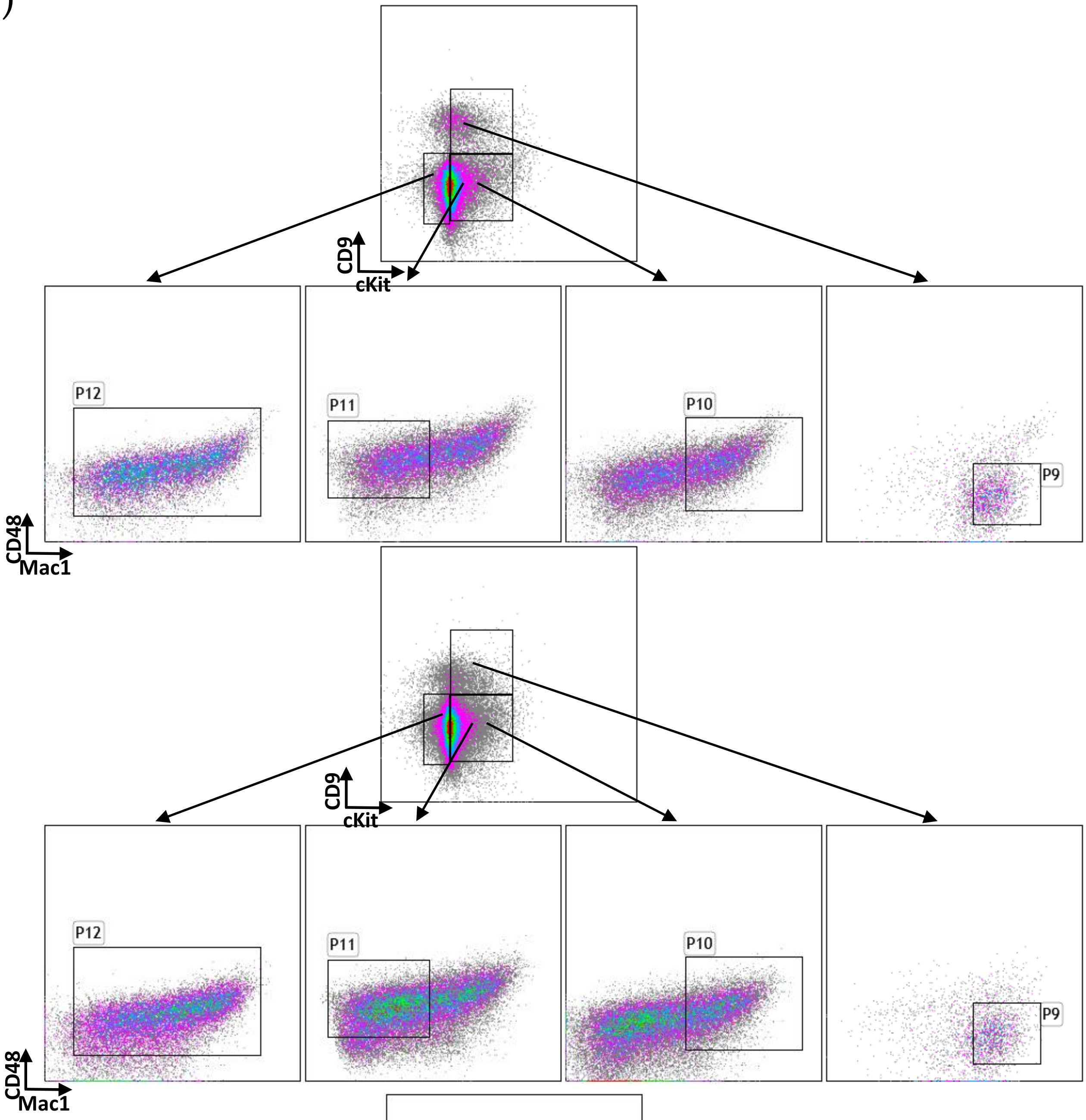
CD9
cKit
P12
P11
P10
P9
CD48
Mac1
CD9
cKit
P12
P11
P10
P9
CD48
Mac1

(e)

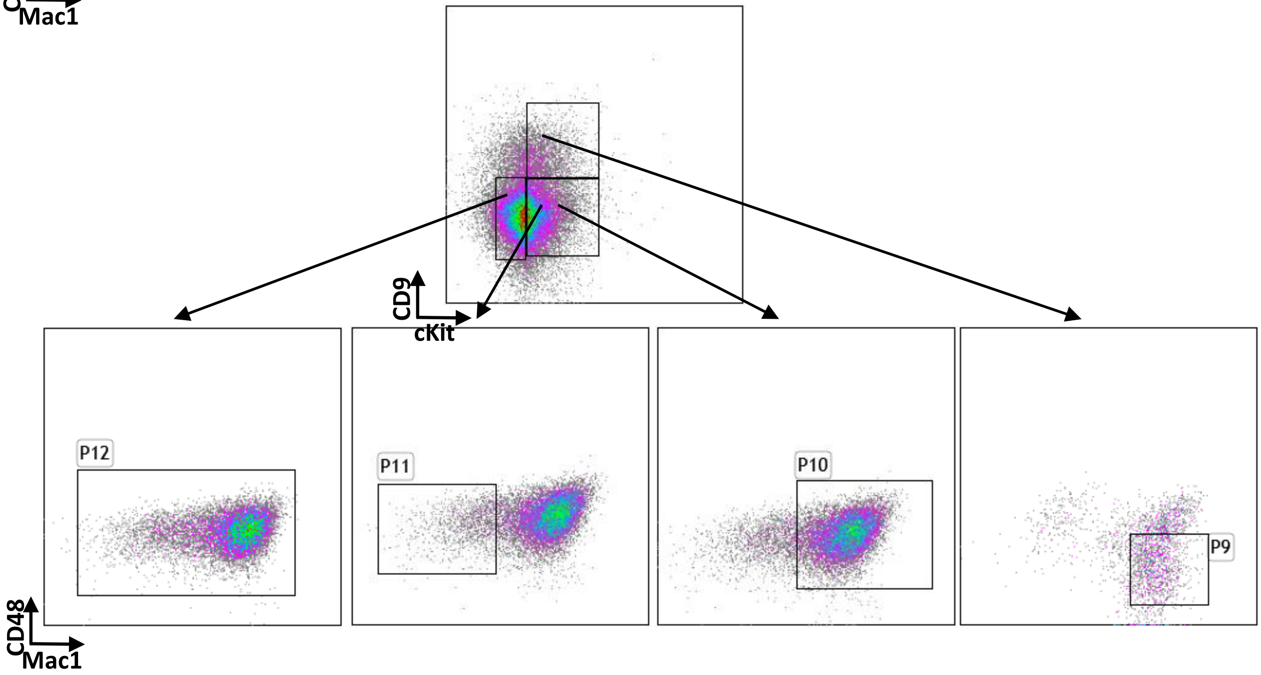
CD9
cKit
P12
P11
P10
P9
CD48
Mac1

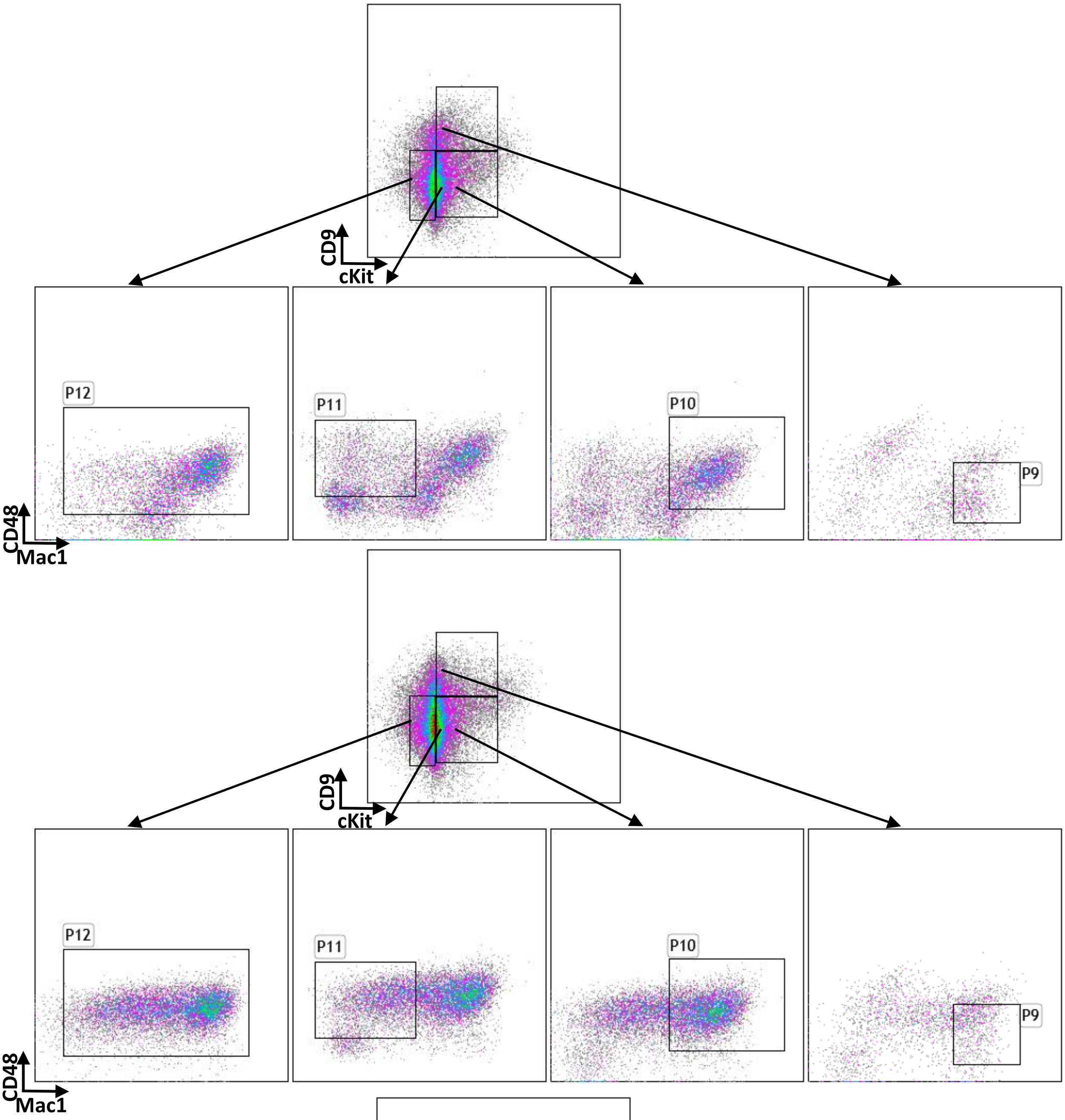
CD9
cKit
P12
P11
P10
P9
CD48
Mac1
CD9
cKit
P12
P11
P10
P9
CD48
Mac1

(f)

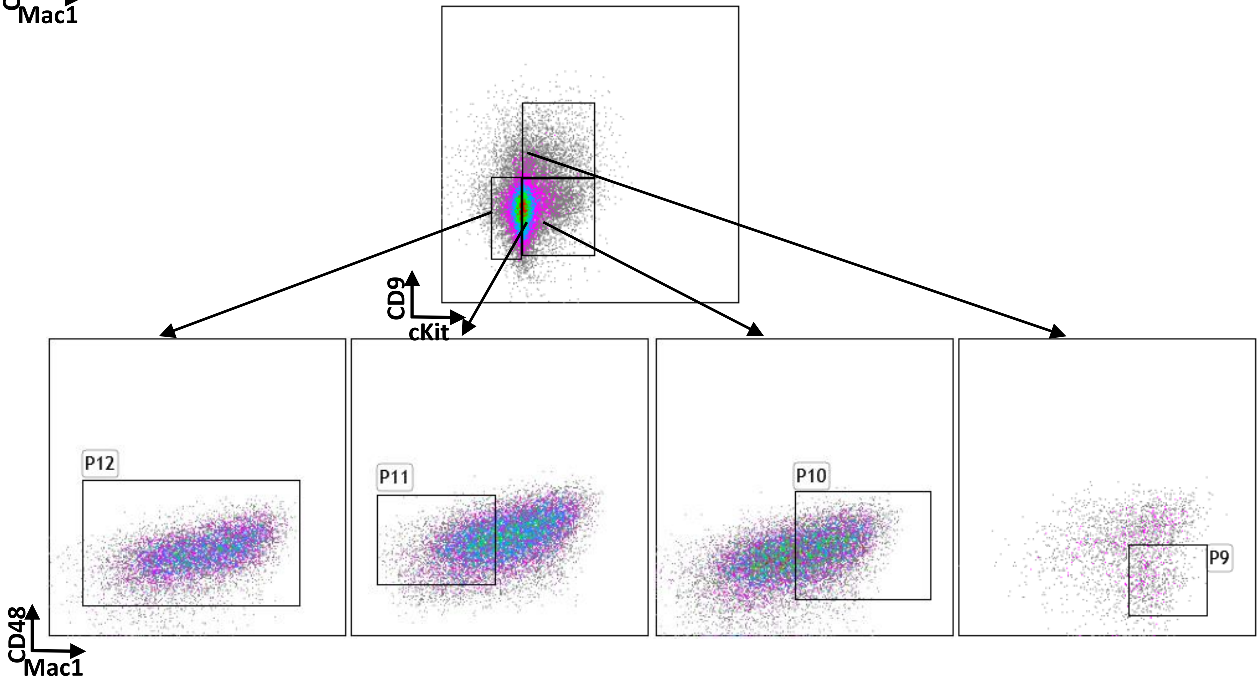
CD9
cKit
P12
P11
P10
P9
CD48
Mac1

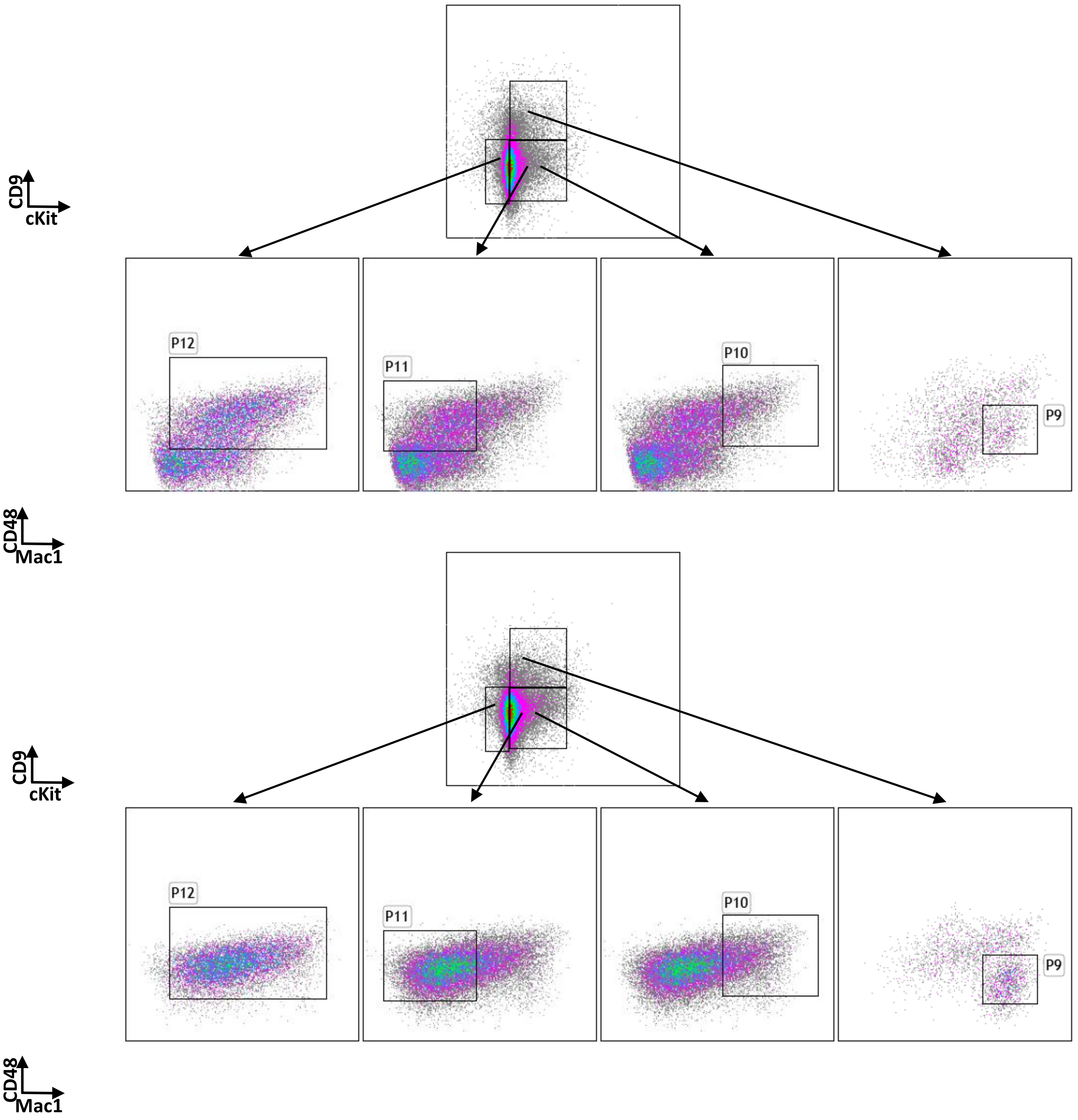

Figure S3. Distribution of the sub- populations of ML23 in various organ

The expression (from Zs+ cells) of each of the four subpopulations (P9-12) as measured by FACS analysis for cells extracted from six different tissues of ML23 leukemic mice (n=3). (**a**). Femur. (**b**). Tibia. (**c**). Pelvis. (**d**). Lymph Nodes. (**e**). Spleen. (**f**). Thymus. Cells were PRE-GATED to ZsGreen+, at least 90% in each sample (not shown).

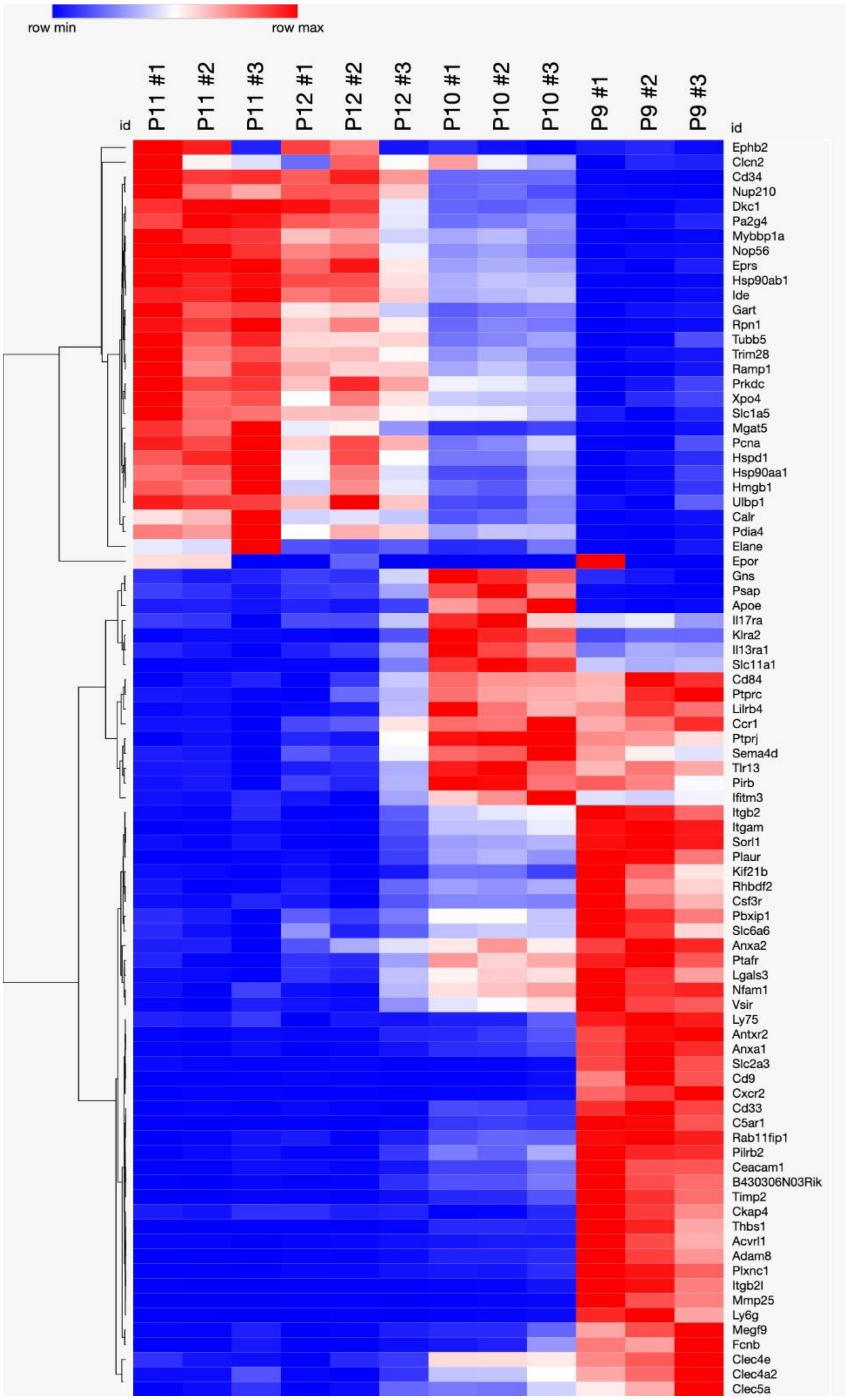

<u>Figure S4. LSC has a unique surface markers pattern:</u> The expression level of membrane protein genes was examined in the subpopulations. 85 genes were found to be enriched or suppressed .
